# Fully Automated Detection of Corticospinal Tract Damage in Chronic Stroke Patients

**DOI:** 10.1155/2014/370849

**Published:** 2014-01-15

**Authors:** Ming Yang, Ya-ru Yang, Hui-jun Li, Xue-song Lu, Yong-mei Shi, Bin Liu, Hua-jun Chen, Gao-jun Teng

**Affiliations:** ^1^Department of Radiology, Zhongda Hospital, Southeast University, Nanjing 210009, China; ^2^Department of Rehabilitation, Zhongda Hospital, Southeast University, Nanjing 210009, China; ^3^Department of Neurology, Affiliated Zhongda Hospital of Southeast University, Nanjing 210009, China

## Abstract

Structural integrity of the corticospinal tract (CST) after stroke is closely linked to the degree of motor impairment. However, current methods for measurement of fractional atrophy (FA) of CST based on region of interest (ROI) are time-consuming and open to bias. Here, we used tract-based spatial statistics (TBSS) together with a CST template with healthy volunteers to quantify structural integrity of CST automatically. Two groups of patients after ischemic stroke were enrolled, group 1 (10 patients, 7 men, and Fugl-Meyer assessment (FMA) scores ⩽ 50) and group 2 (12 patients, 12 men, and FMA scores = 100). CST of FA_ipsi_, FA_contra_, and FA_ratio_ was compared between the two groups. Relative to group 2, FA was decreased in group 1 in the ipsilesional CST (*P* < 0.01), as well as the FA_ratio_ (*P* < 0.01). There was no significant difference between the two subgroups in the contralesional CST (*P* = 0.23). Compared with contralesional CST, FA of ipsilesional CST decreased in group 1 (*P* < 0.01). These results suggest that the automated method used in our study could detect a surrogate biomarker to quantify the CST after stroke, which would facilitate implementation of clinical practice.

## 1. Introduction

Diffusion tensor imaging (DTI) can delineate anatomic connectivity of white matter and evaluate tract disruption in vivo, which is increasingly used in stroke-related research [1–5]. DTI-derived parameter such as fractional anisotropy (FA) has been found to reliably reflect the microstructural status of corticospinal tract (CST) in patients with stroke [6–8]. Greater gains in motor function were related to higher FA values of ipsilesional CST, and slice-by-slice analysis of FA values along the CST demonstrated that the more the ipsilesional FA profiles of patients resembled those of healthy controls, the greater their functional improvement was [6]. Meanwhile the reverse is also true that greater loss of structural integrity of the ipsilesional CST is associated with poorer motor outcomes in patients with hemiparetic stroke [7, 8].

Despite these advances, some factors impede the uptake of these approaches. CST tracking in individual stroke is often difficult due to interruption of fibers by the infarct which can result in the unreliable morphology of the tracts. Moreover, manual placement of regions of interest (ROI) in individual patients is also problematic because of operator bias, and manual labeling is time-consuming. For these reasons, its feasibility is limited. Therefore, a fully automated method of evaluating CST is urgently needed to satisfy the translational potential of CST injury quantification to clinical practice.

Tract-based spatial statistics (TBSS) is a new approach which is fully automated to investigate the whole brain without prespecification of tracts of interest [9]. Meanwhile the method does not require smoothing and performs alignment across participants and has a high sensitivity for identifying white-matter (WM) deficits using nonlinear registration and tract projection. Recently this method has been applied to evaluate white-matter changes of schizophrenia [10, 11] and Parkinson's disease [12]. Yet, few studies have reported stroke-related changes in the WM structural networks using TBSS [13–15]. Most of these studies mirrored lesion in one hemisphere to another across the midsagittal axis. The goal is to increase patients' homogeneity and control for the location of the lesion to be able to interpret voxel-wise analysis [13, 14]. However, this method does not use a symmetric cerebral template and may cause unreliable results. Only one study did not adopt mirror about midline due to enrolling lesions in the same hemisphere [15]. However the lesion locations are not in accordance with clinical practice and greatly limit the subject sample.

The aim of our study was to contrast DTI-derived fully automated detection of corticospinal tract damage between two subgroups of chronic stroke patients with different recovery and meanwhile investigate the possibility of the translational potential of CST injury quantification to clinical practice. Given the diversity issue in the stroke population, a skeletonized mean FA image came from TBSS was not used to contrast between two subgroups and only individual FA images were evaluated in our study. Each participant's (aligned) FA image was acquired by filling the skeleton with FA values from the nearest relevant tract center. Then individual CSTs were acquired by overlaying individual FA images with CST digital WM atlas JHU [16]. This was done to fully automate the process of obtaining ipsilesional and contralesional CST individually with no need to mirror the lesion from one hemisphere to another.

## 2. Methods

### 2.1. Subjects

The study group consisted of 22 patients, all of whom had ischemic strokes in the middle cerebral artery territory at least 6 months before entering the study. The patients were divided into 2 subgroups according to their Fugl-Meyer assessment (FMA) scores: group 1 (10 patients, 7 men, and FMA scores ⩽ 50) and group 2 (12 patients, 12 men, and FMA scores = 100). The FMA is a 50-item motor function assessment with scores ranging from 0 to 100. FMA scores ⩽ 50 belong to severe motor impairment, and FMA scores = 100 means no motor impairment [17]. It is one of the most frequently used clinical motor impairment tests in stroke rehabilitation research. All patients in this study required inpatient rehabilitation, which consisted of standard physical and occupational therapy. Exclusion criteria were as follows: (1) prior or subsequent symptomatic stroke; (2) bihemispheric or brain stem infarcts, primary intracerebral hemorrhages, and other disorders that impaired motor function of the stroke-affected hand and leg; (3) other concomitant neurological or psychiatric diseases; and (4) contraindication to MRI. Patient clinical characteristics and demographic data are summarized in Table 1. This study was approved by the Ethics Committee of Southeast University and a signed informed-consent form was obtained from every subject prior to the experiment.

### 2.2. MRI Procedures

MR images were obtained using a Siemens Verio Tim 3.0 T MR scanner (Siemens Medical Solutions, Erlangen, Germany). DTI images were acquired using a 12-channel phased-array head coil with the implementation of the parallel imaging scheme GRAPPA (GeneRalized Autocalibrating Partially Parallel Acquisitions) and an acceleration factor of 2. A single-shot echo planar imaging (EPI) sequence was used in DTI data including 30 nonlinear diffusion directions with *b* = 1000 s/mm^2^ and an additional volume with *b* = 0 s/mm^2^. The detailed parameters are as follows: number of axial sections, 70; slice thickness, 2 mm; gap, none; acquisition matrix, 128 × 124; TR, 10,900 ms; TE, 95 ms; field of view, 256 mm × 256 mm; and average, 2. We also acquired 3D high resolution brain structural images (voxel size = 1 mm^3^, isotropic) using a T1-weighted MPRAGE sequence for each subject. The sequence parameters were TR/TE = 1900 ms/2.48 ms, inversion time (TI) = 900 ms, flip angle = 9°, FOV = 256 mm × 256 mm, slice thickness = 1 mm, and 176 sagittal slices covering the whole brain. To identify the location and size of the lesion, we acquired fluid attenuated inversion recovery (FLAIR) images: number of axial sections, 20; slice thickness, 5 mm; gap, none; acquisition matrix, 128 × 124; TR, 8,500 ms; TE, 94 ms; field of view, 230 mm × 208 mm; flip angle, 150°. All subjects were scanned using the same MR scanner.

### 2.3. Lesion Mapping

We used MRIcron to define the lesions to obtain a VOI (volume of interest) in the raw T1-weighted images (T1WI). For each patient, we manually outlined the lesion area on each slice while using the FLAIR images as an additional guide to confirm the extent of the lesion. Lesions were defined as hypointensity area with clear border in T1WI. The whole lesion volumes were determined by integration across all relevant slices. Lesions were drawn by an experienced rater who was blinded to the patients' FMA scores.

### 2.4. DTI Data Processing

**Figure 1:**
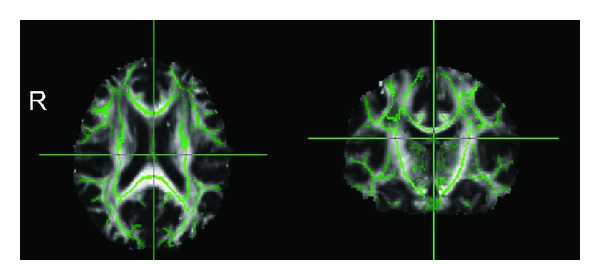
Axial and coronal views of mean FA skeleton which represent the centers of all tracts common to group 1 and group 2.

DTI processing was performed using the FSL 5 (FMRIB Software Library, http://www.fmrib.ox.ac.uk/fsl). Then, a statistical analysis of FA data was carried out using TBSS, part of FSL [9]. First, for each participant, DTI images were registered to the corresponding *b* = 0 images with an affine transformation to correct eddy-current distortion and head motion by using FMRIB's Diffusion Toolbox (FDT, part of FSL). Next, no brain tissues were removed using the Brain Extraction Tool (BET, part of FSL) [18]. These were referred to as the preprocessing stages. For the next step, the FA data were aligned into 1 mm × 1 mm × 1 mm Montreal Neurological Institute (MNI) 152 space using the FMRIB's Nonlinear Image Registration Tool (FNIRT). Then, the mean FA image (threshold of 0.2) was created and thinned to create a mean FA skeleton which represents the centers of all tracts common to group 1 and group 2 (Figure 1). Each subject's aligned FA data was then projected onto this skeleton and the skeletonized FA images were obtained.

### 2.5. ROI Analysis

Our goal is to calculate regional FA for fiber tracts defined in the JHU white-matter tract template [16]. JHU white-matter tract template is defined in the MNI152 space. For each skeletonized FA map, we used the JHU WM tract template to delineate bilateral CSTs. Ipsilateral FA (FA_ipsi_), contralateral FA (FA_contra_), and FA_ratio_ were computed in terms of three measures over the whole CST, FA_ratio_ was computed as a ratio (FA_ipsi_/FA_contra_).

### 2.6. Statistical Analysis

Statistical analysis of the demographic and clinical data was performed using the SPSS 16.0 (SPSS Inc., Chicago, IL, USA). The threshold for statistical significance was *P* < 0.05. The differences between the two groups in age, education, and the time after stroke were tested with two sample *t*-tests, as well as FA_ipsi_, FA_contra_, and FA_ratio_ of CST. Fisher's exact test was used to assess gender difference and side of lesion. Data were normally distributed according to Kolmogorov-Smirnov test. 

## 3. Results

### 3.1. Clinical Data

No significant differences between the two subgroups were observed in age (group 1: mean ± SD, 61.10 ± 10.28 years; group 2: mean ± SD, 59.00 ± 7.91 years; *P* = 0.59), time after stroke (group 1: mean ± SD, 28.12 ± 19.78 months; group 2: mean ± SD, 20.03 ± 14.40 months; *P* = 0.28), education (group 1: mean ± SD, 8.40 ± 4.35 years; group 2: mean ± SD, 9.67 ± 4.10 years; *P* = 0.49), lesion volume (group 1: mean ± SD, 1690.6 ± 1293.2 mm^3^; group 2: mean ± SD, 2539.6 ± 7433.4 mm^3^; *P* = 0.72), sex (group 1: M/F, 7/3; group 2: M/F, 12/0; *P* = 0.08), and side (group 1: L/R, 3/7; group 2: L/R, 9/3; *P* = 0.08). We found a significant difference of the FMA score between the 2 subgroups (group 1: mean ± SD, 31.10 ± 13.12; group 2: mean ± SD, 100 ± 0 months; *P* < 0.01).

### 3.2. ROI Analysis of FA of CST

FA of CST between the two subgroups can be seen in Table 2. Compared with group 2, FA was decreased in group 1 in the ipsilesional CST (*P* < 0.01), as well as the FA_ratio_ (*P* < 0.01). There was no significant difference between the two groups in the contralesional CST (*P* = 0.23). Compared with contralesional CST, FA of ipsilesional CST decreased in group 1 (*P* < 0.01). There was no significant difference between bilateral CSTs in group 2.

## 4. Discussion

**Figure 2:**
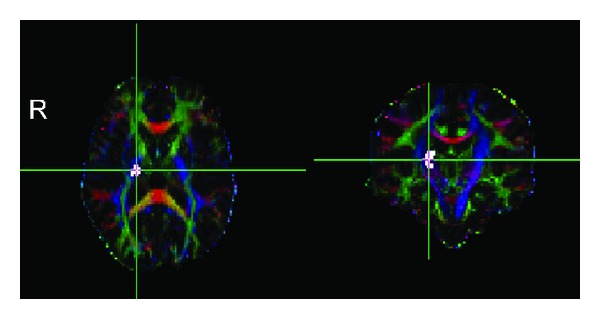
Axial and coronal views of individual color-coded FA map of subject 01 from group 1 together with lesion map. The patient's FMA score was 42 and the time after stroke was 9.7 months. Colors indicate direction of fiber tracts (red, left-right; blue, craniocaudal; green, anterior-posterior). Crosshairs are centered on the infarct (white color) which shows that right posterior limb of internal capsule (part of CST) was interrupted by the lesion.

**Figure 3:**
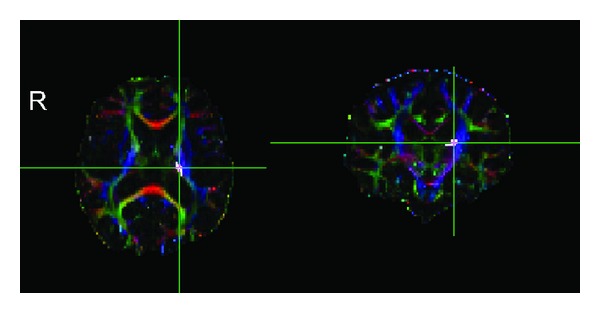
Axial and coronal views of individual color-coded FA map of subject 05 from group 2 together with lesion map. The patient's FMA score was 100 and the time after stroke was 43 months. Colors indicate direction of fiber tracts (red, left-right; blue, craniocaudal; green, anterior-posterior). Crosshairs are centered on the infarct (white color) which shows that the lesion is close to the left posterior limb of internal capsule and CST is intact.

To the best of our knowledge, this study is the first that combines TBSS and CST templates to quantify CST in subgroups of patients who have had a stroke with different recoveries. It is well established that quantitative detection of CST based on DTI plays an important role in recovery after stroke. Greater gains in motor function were related to higher FA values of ipsilesional CST [6], and greater loss in structural integrity of the ipsilesional CST is associated with poorer motor outcomes in patients with hemiparetic stroke [7, 8]. In our study, we enrolled two groups of patients with poor (group 1, Figure 2) and good recoveries (group 2, Figure 3) to validate the automated method in evaluating injury of CST. In accordance with previous studies, we found that FA of the ipsilesional CST significantly decreased in group 1 compared with group 2. And FA_ratio_ also decreased in group 1 compared with group 2.

In addition, we found that there is no significant change of FA in the contralesional CST between the two subgroups. This result was in accordance with the other studies, where FA values of contralateral pyramidal tracts (PT) did not differ from the values contrasted with control [1, 6]. However, there were some inconsistent findings. Puig et al. showed that mean FA values in unaffected CST increased at day 30 in line with increasing motor deficit [2]. Another study found that the FA value of CST in the unaffected hemisphere in patients who could not walk independently was significantly lower than that of the control group (*P* < 0.05) [19]. This discrepancy may be due to the differences in the detailed methodology including the variable subjects and sample, function recovery standard scale, and data processing. The exact FA value change of contralesional CST needs further evaluation based on a larger sample size, a longitudinal evaluation, and more accurate measurement.

Our study adopted a method of both TBSS and a CST template to quantify CST fully automatically in subgroup stroke patients. It has the advantage of TBSS, which eliminates the need for smoothing and alignment across participants, as well as prespecification of tracts of interest. Meanwhile a CST template from healthy volunteers was referenced and bilateral CSTs of individual patients can be fully automatically obtained through projecting onto this skeleton. The method is automatic and objective, improving efficiency and eliminating bias, and would facilitate implementation in clinical practice.

Our study had some limitations. First, the spatial accuracy of TBSS analysis is limited by the skeleton and, therefore, does not provide significant details in some locations. However we carefully examined the result of normalization for each participant to confirm the accuracy and validity of our postprocessing. Second, this is a cross-sectional study with relatively small sample size, and a large scale, longitudinal evaluation is needed to further confirm the reliability of our method, as well as the occurrence and development roles of CST. Finally, we did not consider the extent of leukoaraiosis, which will be involved in a future study.

In conclusion, our research demonstrated the possibility to automatically quantify CST, opening the avenue for large-scale studies of the utility of unbiased assessment of CST integrity after stroke in predicting motor outcomes.

## Figures and Tables

**Table 1:** Clinical and demographic data of 22 patients with ischemic stroke.

Patient no.	Sex	Age (y)	Dominant hand	Location of lesion	Time after stroke (months)	Education (years)	Lesion volume (mm^3^)	FMA Score
Group 1								
01	M	67	R	R, PLIC	9.7	9	551.2	42
02	F	64	R	L, CR, temporal lobe	6.5	8	859.3	31
03	M	63	R	R, PLIC, parietal lobe	23.3	6	1914.0	10
04	M	37	R	R, PLIC, BG	6.9	17	1429.6	30
05	M	51	R	R, PLIC, BG	59	12	1960.8	47
06	M	65	R	R, CR, BG	54.4	9	1339.0	42
07	M	60	R	R, CR, BG	42.2	9	5133.6	47
08	F	69	R	L, CR, BG	12.6	8	1280.8	20
09	M	62	R	L, BG, CR	26.2	6	1592.6	17
10	F	73	R	R, BG, CR	40.4	0	845.0	25
Group 2								
01	M	56	R	R, TH	12.2	8	25.7	100
02	M	52	R	L, CR, CS	7.2	12	100.1	100
03	M	60	R	L, CR, temporal lobe	11	12	26071.5	100
04	M	43	R	L, CR, BG	16	9	84.9	100
05	M	70	R	L, TH, BG	43	12	624.7	100
06	M	70	R	L, TH	25.3	6	371.0	100
07	M	56	R	L, BG	24	12	61.0	100
08	M	59	R	L, CR, BG	53.4	12	2073.3	100
09	M	60	R	L, BG	10.6	12	29.6	100
10	M	61	R	R, TH	12.7	0	96.3	100
11	M	68	R	L, CR, BG	9.7	6	772.5	100
12	M	53	R	R, BG	15.2	15	164.0	100

M: male; F: female; L: left; R: right; IC: internal capsule; PLIC: posterior limb of IC; BG: basal ganglia; CR: corona radiata; TH: thalamus; CS: centrum semiovale.

**Table 2:** FA of CST between the two subgroups.

	FA_ipsi_	FA_contra_	FA_ratio_	*P* value (FA_ipsi_−FA_contra_)
Group 1	0.14 ± 0.01	0.17 ± 0.01	0.83 ± 0.07	<0.01
Group 2	0.17 ± 0.007	0.17 ± 0.009	0.98 ± 0.04	0.24
*P* value	<0.01	0.23	<0.01	

Note: data are means ± SD.
